# The Hypolipidemic Characteristics of a Methanol Extract of Fermented Green Tea and Spore of *Eurotium cristatum* SXHBTBU1934 in Golden Hamsters

**DOI:** 10.3390/nu15061329

**Published:** 2023-03-08

**Authors:** Fuhang Song, Kai Zhang, Jinpeng Yang, Annette S. Wilson, Caixia Chen, Xiuli Xu

**Affiliations:** 1 School of Light Industry, Beijing Technology and Business University, Beijing 100048, China; zhangkai2030302071@st.btbu.edu.cn; 2 School of Ocean Sciences, China University of Geosciences, Beijing 100083, China; yangjinpeng@cugb.edu.cn; 3 School of Medicine, University of Pittsburgh, Pittsburgh, PA 15213, USA; aswilson@pitt.edu (A.S.W.); cac321@pitt.edu (C.C.)

**Keywords:** hypolipidemic characteristics, *Eurotium cristatum*, Fuzhuan brick tea, methanol extract, spore, alkaloid

## Abstract

Fuzhuan brick tea (FBT), a distinctive Chinese dark tea with the predominant fungus of *Eurotium cristatum*, offered significant health benefits to Chinese people. In the current study, the in vivo bioactivities of *E. cristatum* (SXHBTBU1934) fermented green tea and spores of *E. cristatum* fermented on wheat were investigated, respectively. The methanol extract of fermented green tea and spore of *E. cristatum* both showed potent lipid-lowering activity in the blood of a high-fat diet induced hyperlipidemia model in golden hamsters and significantly reduced the accumulation of fat granules in the liver. These results indicated that the key active components were produced by *E. cristatum*. Chemical investigations suggested similar components in the two extracts and led to the identification of a new alkaloid, namely variecolorin P (**1**), along with four known structurally related compounds, (-)-neoechinulin A (**2**), neoechinulin D (**3**), variecolorin G (**4**), and echinulin (**5**). The structure of the new alkaloid was elucidated by HRESIMS, ^1^H, ^13^C, and 2D NMR analysis. The lipid-lowering activity of these compounds was evaluated using an oleic acid-induced HepG2 cell line model. Compound **1** significantly reduced the lipid accumulation in the HepG2 cell line with an IC_50_ value of 0.127 μM.

## 1. Introduction

The continuous ingestion of a high-fat diet and decreased energy expenditure cause the excessive accumulation of fat, which lead to chronic diseases such as hepatic steatosis, cardiovascular diseases, and type 2 diabetes [1,2,3]. Obesity has become a widespread public health issue all over the world, most of which comes from the excessive accumulation of fat [4]. To treat obesity and avoid related diseases, increasing amounts of studies focused on exploring a new dietary effective functional food, to slow down fatty liver disease and regulate or improve intestinal microbiota with limited side effects [5].

Fuzhuan brick tea (FBT) is a popular beverage in China due to its unique flavor and variety of health-promoting functions. FBT or metabolites isolated from FBT exhibited a variety of bioactivities against metabolic diseases, including regulating the expression of multiple genes which mediated regulation of blood lipids [6,7,8,9,10,11], preventing obesity [12,13,14], hyperlipidemia [15] and hyperglycemia [10,16,17], ameliorating colitis [18], anti-bacterial [19], anti-oxidation [20], and modulating gut microbiota [21]. *Eurotium cristatum*, the predominant microorganism in FBT, was considered a probiotic that can alleviate the obesity of rodents induced by a high-fat diet [12]. The theabrownins from dark tea fermented by *E. cristatum* PW-1 were proved to show hypolipidemic activity in high-fat zebrafish [22]. The active metabolites secreted by *E. cristatum* may enhance the human immune system [23]. Further investigation of biomolecules from the spore of *E. cristatum* will promote the development of new functional foods.

Traditionally, FBT is cooked with milk or butter, which suggests that both water-soluble and lipid-soluble constituents could be extracted. As methanol is a good organic solvent to extract most of the polar and nonpolar compounds, in this study, it was used for extraction. The lipid-lowering activities of the methanol extract of *E. cristatum* fermented green tea and spore from *E. cristatum* fermented wheat were investigated, respectively in a high-fat-diet-induced hyperlipidemia model in golden hamsters. Furthermore, the chemical composition and bioactivity of the extracts were investigated.

## 2. Materials and Methods

### 2.1. Fungal Material, FBT, Spore of E. cristatum, and Extraction

Strain SXHBTBU1934 was isolated from FBT, bought from the market of Xixian New District, Shaanxi Province, China, and grown on a potato dextrose agar plate at 28 °C for 7 days. The genomic DNA of SXHBTBU1934 was extracted using DNA quick Plant System (Tiangen). The Internal Transcribed Spacer (ITS) sequence was amplified by using a conventional primer pair of ITS4 (5′-TCCTCCGCTTATTGATATGC-3′) and ITS5 (5′-GGAAGTAAAAGTCGTAACAAGG-3′). PCR products were sequenced by Beijing Qingke Biotechnology Co., Ltd. (Beijing, China). Strain SXHBTBU1934 was identified as *E. cristatum* based on gene sequence analysis of ITS. The strain was deposited at the Beijing Technology and Business University, Beijing, China.

Strain SXHBTBU1934 was inoculated on ten potato dextrose agar plates and cultured for 5 days at 28 °C. In total, 10 mL of distilled water was added to the plate to wash out the spore (3 times), and the spore suspension was combined as seed. In total, 5 mL of the seed was inoculated into twenty 1000 mL conical flasks, each containing 100 g green tea and 30 mL distilled water which was sterilized for 15 min at 121 °C. The inoculated flasks were incubated stationary at 28 °C for 20 days. The fermented tea by SXHBTBU1934 was dried by air. In total, 2 kg of dried fermented tea was soaked in 5 L methanol for 24 h and extracted three times. The organic solvent was evaporated in vacuo at 45 °C to yield a brown crude extract, which was used for HPLC-MS analysis and in vivo experiments.

For comparison, 10 g fermented tea by SXHBTBU1934 was added to 100 mL fresh milk and boiled for 2 min. Then, 1 mL methanol was added to a 2 mL aliquot of the milk extract and centrifuged (10,000 rpm) for 3 min. In total, 1 mL of supernatant was filtered by a 0.45 µm filter for HPLC analysis. The green tea without fermentation was also extracted with methanol.

Spores of *E. cristatum* were collected by Bio-tea Co., Ltd. of Shaanxi Biotech Group (Xianyang, China). *E. cristatum* was fermented on wheat on a large scale and the spores were collected as mentioned above. Briefly, the fermented wheat was washed three times with distilled water and the water was combined to collect the spore suspension, which was then centrifuged at 4000 rpm for 20 min. The supernatant was discarded and the spore pellet was dried by vacuum freeze-drying.

### 2.2. HPLC-MS Analysis of Extracts

The HPLC-MS analysis was performed on an Agilent 1200-MSD HPLC-MS system with reversed-phase Agilent Eclipse XDB-C8 column (4.6 mm i.d. × 150 mm, 5 μm, Agilent, Santa Clara, CA, USA) at 28 °C. Mobile phase A consisted of ultrapure water containing 0.1% methanolic acid, and mobile phase B was 100% acetonitrile. Gradient elution was performed by 10–100% B with a linear change within 15 min, then 100% acetonitrile for 5 min. Absorbance was monitored at 254 nm.

### 2.3. Compound Isolation

The crude extract from the spore of *E. cristatum* was dissolved in MeOH at a concentration of 100 mg/mL. The sample was subjected to preparative HPLC using a reversed-phase Agilent Eclipse XDB-C8 column (9.4 mm i.d. × 250 mm, 5 μm, Agilent, Santa Clara, CA, USA) at 28 °C, with a gradient elution of MeCN in H_2_O from 40–80% within 20 min to yield compounds **1**–**5**.

### 2.4. Structure Determination

NMR spectra were obtained on a Bruker Avance 500 spectrometer at 25 °C (operating at 500 MHz for ^1^H-NMR, 125 MHz for ^13^C-NMR) with residual solvent peaks as references (CDCl_3_-*d*_6_: *δ*_H_ 7.26, *δ*_C_ 77.16). High-resolution electrospray ionization mass spectrometry (HR-ESIMS) measurements were obtained on an Accurate-Mass-Q-TOF LC/MS 6520 instrument (Santa Clara, CA, USA) in the positive ion mode. Optical rotations were measured on a Perkin-Elmer Model 343 polarimeter. Compound structures were elucidated by analyzing ^1^H NMR, ^13^C NMR, Heteronuclear Multiple Bond Correlation (HMBC), Heteronuclear Single Quantum Correlation (HSQC), ^1^H-^1^H Correlation Spectroscopy (COSY), and comparison with the previously reported data. The structure formula was finally confirmed by HR-ESIMS.

### 2.5. Animal Experimental Design

In total, 50 healthy specific pathogen-free male golden hamsters (6 weeks old with a similar body weight of 100 g) were purchased from Beijing Vital River Laboratory Animal Technology Co., Ltd. (Beijing, China). Hamsters were maintained in a temperature-controlled room (23 °C) with a 12 h:12 h light–dark cycle (lights on from 8:00 to 20:00) and allowed one week acclimatization period [24]. Deionized water was provided continuously for hamsters to drink freely. All procedures using hamsters in this study were conducted by the recommendations in the Guide for the Care and Use of Laboratory Animals of the People’s Republic of China. Golden hamsters were allowed one week of the adaptation period. Then, the hamsters were divided into two groups, which included 10 and 40 hamsters, respectively. In total, 10 hamsters were fed a normal diet as the control group (ND), and 40 hamsters in the experiment group were fed a high-fat diet (HFD) for rapid body weight growth. After 2 weeks, 30 hamsters from the HFD group with higher body weight (weight gain > 8 g) were selected and divided into 3 groups: high-fat diet control group (HFD), high-fat diet with methanol extract of fermented green tea intervention group (MET), and high-fat diet with the spore intervention group (ST). The dried methanol extract of fermented green tea and the dried spore of *E. cristatum* were both dissolved or suspended in 0.5% boxymethylcellulose sodium (80 mg/mL) for the in vivo experiment. The hamsters in MET and ST groups were intragastrically administered 400 mg of dissolved fermented green tea extract or spore suspension per kg body weight (5 mL/kg body weight), once daily for 2 weeks. The hamsters in the ND and HFD groups were administered 0.5% boxymethylcellulose sodium in the same way. After the last administration, all of the hamsters fasted for 12 h [25,26].

### 2.6. Serum and Liver Collection/Biochemical Analyses

Blood was collected from the retro-orbital plexus after the hamsters fasted for 12 h. The blood samples were centrifuged at 3000 rpm for 5 min to separate the serum for further biochemical analyses. Sections of fresh liver were put into a glass homogenizer and homogenized with 9 times (*w*/*w*) of physiological saline. The homogenized liver was centrifuged at 3000 rpm for 5 min and the supernatant was collected for further analysis. Total cholesterol (TC), triglyceride (TG), high-density lipoprotein cholesterol (HDL-C), and low-density lipoprotein cholesterol (LDL-C) levels in serum and liver, glucose, insulin, leptin, and free fatty acids (FFA) levels in serum were analyzed using commercial kits following the manufacturer’s instructions. Free fatty acid (FFA) assay kit, mouse insulin ELISA kit, and mouse leptin ELISA kit were purchased from Beijing Solarbio Science & Technology Co, Ltd., Beijing, China. Mouse glucose assay kit, total cholesterol (TC) content assay kit, triglyceride (TG) content assay kit, HDL-cholesterol assay kit, and LDL-cholesterol assay kit were purchased from BioSino Bio-Technology & Science Inc., Beijing, China.

### 2.7. Cell Culture and Treatment

Human hepatoma HepG2 cells were obtained from the Beijing Union Medical Cell Resource Center (Basic Medical Cell Center, Institute of Basic Medical Sciences, Chinese Academy of Medical Sciences). The cells were cultured in DMEM containing 10% FBS and 1% penicillin–streptomycin and incubated with 5% CO_2_ at 37 °C. Cells were allowed to grow to approximately 80% confluency and were sub-cultured at a ratio of 1:3 [27]. In this study, to simulate the process of nonalcoholic fatty liver disease in vitro, 0.2 mM oleic acid (OA) was added to the culture medium for 48 h to induce human HepG2 to establish a nonalcoholic fatty liver cell model [28].

### 2.8. Cell Viability Assay

HepG2 cells were seeded into 96-well plates after achieving logarithmic growth and cultured overnight at 37 °C with 5% CO_2_. After treatment with different concentrations of compounds (dissolved in DMSO) for 24 h, 10 μL CCK-8 solution (Cell Counting Kit-8, Sigma-Aldrich (St. Louis, MO, USA)) was added to each well and the plate was incubated for 2 h in the incubator. Then, the absorbance at 450 nm was measured using a microplate reader. The normal control group was treated with the same amount of DMSO as the experiment group [29]. Cell viability was calculated by: cell viability (%) = (OD_drug_/OD_control_) × 100%.

### 2.9. Bodipy Staining

After treatment with compounds for 24 h in a 96-well plate, the supernatant of the HepG2 cells was discarded and cells were washed twice with PBS, then the cells were fixed with 4% paraformaldehyde at room temperature for 20 min. After removing the fixative reagent, the cells were washed twice with PBS. HepG2 cells were stained with 100 μL Bodipy (2 μg/mL, Thermo Fisher Scientific (Waltham, MA, USA)) in the dark at 25 °C for 30 min [30]. The fluorescence was measured using a fluorescence plate reader (Ex = 500 nm, Em = 550 nm, POLARstar, BMG Labtech (Offenburg, Germany)) [29]. The inhibition rate was calculated by comparison with the control group which was just treated with DMSO.

### 2.10. Oil Red O Staining

HepG2 cells were treated with different concentrations of compounds for 24 h, then were washed twice with PBS and fixed with 4% paraformaldehyde for 15–20 min. The cells were washed with PBS and stained with Oil Red O solution (50 µL per well, Sigma-Aldrich) at 25 °C for 1 h. After washed with PBS for 3–5 times, the lipid droplets and cell morphology were observed by a light microscope and photographed [29]. The area of lipid droplets was statistically compared for IC_50_ calculation with the control group which was treated with DMSO using Image J software.

### 2.11. Statistical Analysis

Statistical analyses were performed using GraphPad Prism 8.0.2 (GraphPad Software, San Diego, CA, USA). The statistical significance of differences between groups was calculated by ordinary one-way ANOVA. The experimental data are presented as mean ± standard deviations (SD). Statistical significance is denoted by * for *p* < 0.05, ** for *p* < 0.01, *** for *p* < 0.001, and **** for *p* < 0.0001.

## 3. Results

### 3.1. Spores of E. cristatum Share Similiar Components as E. cristatum Fermented Green Tea

**Figure 1:**
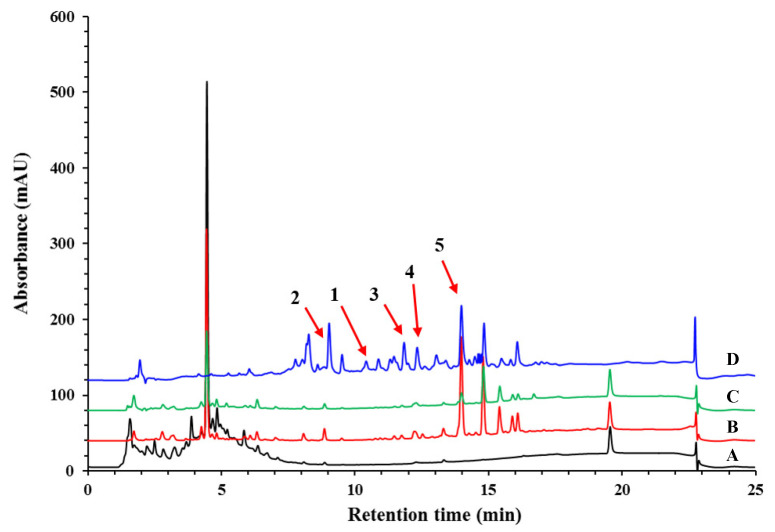
Component comparison of methanol extract of spores, methanol extracts of green tea with and without fermentation, and milk extract of fermented green tea. **A**: methanol extract of green tea (black); **B**: methanol extract of fermented green tea (red); **C**: milk extract of fermented green tea (green); **D**: methanol extract of spore (blue). **1** (variecolorin P); **2** ((-)-neoechinulin A); **3** (neoechinulin D); **4** (variecolorin G); **5** (echinulin).

As FBT is traditionally cooked with butter or milk, we investigated the difference between the milk and methanol extracts of *E. cristatum*-fermented green tea, as well as the methanol extract of the spores. The extracts were analyzed by HPLC with a reversed-phase column. As shown in Figure 1, after fermentation by *E. cristatum,* milk, and methanol extracts showed several low-polarity HPLC peaks between the retention times of 13–18 min compared with the extract of green tea without fermentation. For the methanol extract of spore, more peaks with retention times between 7 and 13 min were found. As this extract shows an abundance of compounds, it was selected for further investigation for compound isolation.

### 3.2. Methanol Extract of Fermented Green Tea, as Well as Spore Suspension Alleviated HFD-Induced Body Weight and Ratio of Liver Weight to Body Weight

**Figure 2:**
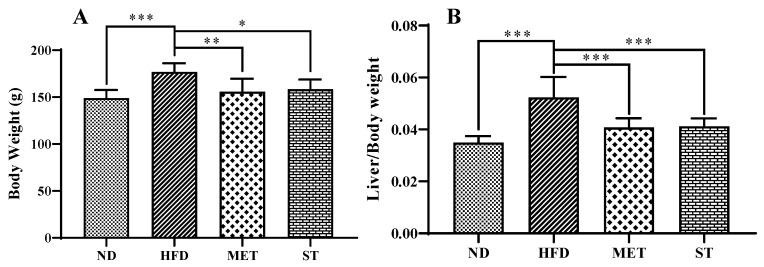
Effects of methanol extract of fermented green tea and spore suspension on body weight of hamsters for 2 consecutive weeks. (**A**): body weight; (**B**): ratio of liver weight to body weight. ND: normal diet treated group; HFD: high-fat diet; MET: high-fat diet with methanol extract of fermented green tea intervention group; ST: high-fat diet with the spore intervention group. Data are expressed as mean ± SD (*n* = 10). * *p* ≤ 0.05; ** *p* ≤ 0.01; and *** *p* ≤ 0.001.

The methanol and milk extracts of fermented green tea shared similar HPLC peaks, while the methanol extract of spores of *E. cristatum* contained more different peaks than the other two. Therefore, we selected the methanol extract of fermented green tea and spore for further investigation. The in vivo effects of the methanol extract of fermented green tea and the spore suspension on body weight and liver weight were studied in a high-fat induced hyperlipidemic model in golden hamsters. After 2 weeks of treatment with a high-fat diet, the body weight of golden hamsters in the HFD group increased to 139.3 ± 6.9 g, which showed a significant difference compared with the NC group (128.8 ± 5.6 g). Interestingly, after following 2 weeks of 400 mg/kg of the methanol extract of fermented green tea or spore suspension intervention, body weight was significantly controlled in groups of MET (155.7 ± 13.8 g, *p* < 0.01) and ST (158.5 ± 10.4 g, *p* < 0.05) (Figure 2A) while the body weight of the high-fat diet control group increased to 176.9 ± 9.2 g. The ratios of liver weight to body weight of hamsters in the MET and ST groups were significantly decreased to 0.0408 ± 0.0035 and 0.0411 ± 0.0031, respectively, *p* < 0.001 compared with the HFD group (0.0524 ± 0.0078). These results showed that the methanol extract of fermented green tea and spore suspension of *E. cristatum* were both able to inhibit the increase of body weight and the ratio of liver weight to body weight in golden hamsters.

### 3.3. Effect of Methanol Extract of Fermented Green Tea and Spore Suspension of E. cristatum on Lipid Levels in Serum and Liver

**Figure 3:**
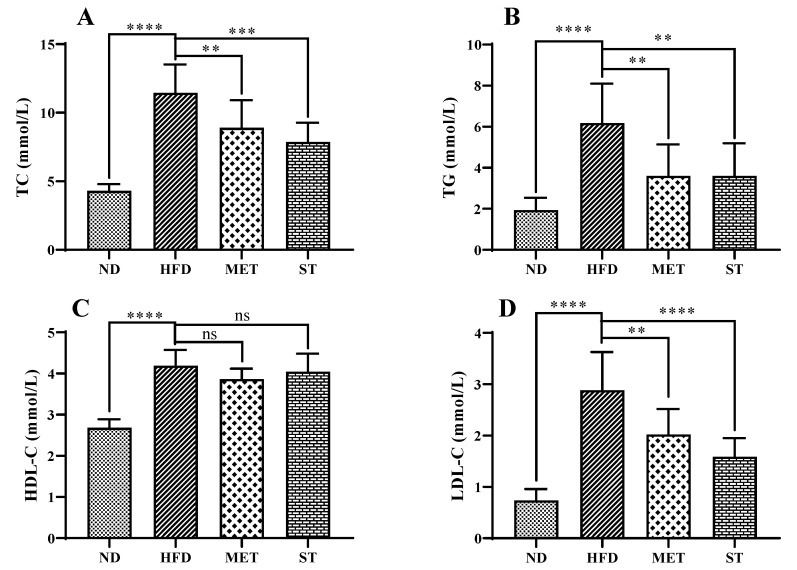
Effects of methanol extract of fermented green tea and spore suspension on TC (**A**), TG (**B**), HDL-C (**C**), LDL-C (**D**) levels in the serum of hamsters for 2 consecutive weeks of intervention. Statistical significance of differences between groups was determined using Ordinary one-way ANOVA. Data are expressed as mean ± SD (*n* = 10). ** *p* ≤ 0.01; *** *p* ≤ 0.001; and **** *p* ≤ 0.0001. “ns” indicates not significant (*p* > 0.05).

**Figure 4:**
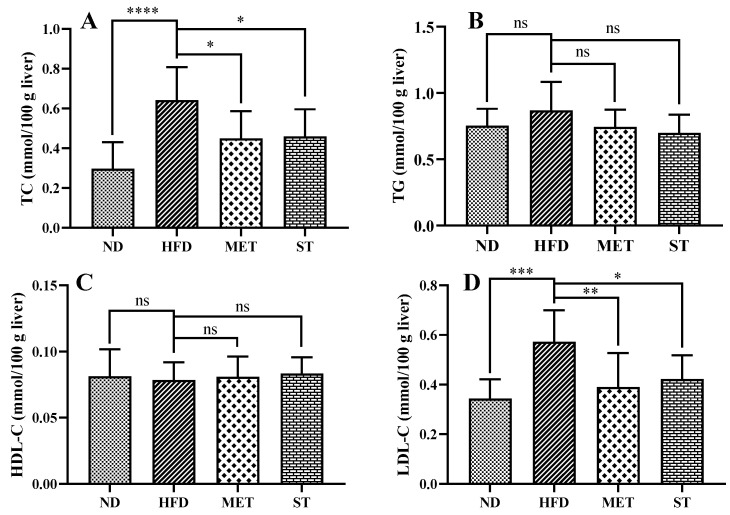
Effects of methanol extract of fermented green tea and spore suspension on TC (**A**), TG (**B**), HDL-C (**C**), LDL-C (**D**) levels in the liver of hamsters for 2 consecutive weeks of intervention. Statistical significance of differences between groups was determined using Ordinary one-way ANOVA. Data are expressed as mean ± SD (*n* = 10). * *p* ≤ 0.05; ** *p* ≤ 0.01; *** *p* ≤ 0.001; and **** *p* ≤ 0.0001. “ns” indicates not significant (*p* > 0.05).

The TC, TG, HDL-C, and LDL-C levels in the serum of golden hamsters for each group are shown in Figure 3. After four weeks of HFD induction, serum lipid levels, including TC, TG, HDL-C, and LDL-C, significantly increased from 4.30 ± 0.50, 1.94 ± 0.60, 2.68 ± 0.21, and 0.74 ± 0.22 mmol/L to 11.45 ± 2.08, 6.18 ± 1.92, 3.87 ± 0.25 and 2.89 ± 0.74 mmol/L, respectively (all groups with *p* < 0.0001). Methanol extract from fermented green tea treatment caused a significant decrease in TC (8.89 ± 2.01 mmol/L, *p* < 0.01), TG (3.60 ± 1.53 mmol/L, *p* < 0.01), and LDL-C (2.02 ± 0.50 mmol/L, *p* < 0.01) levels in comparison with HFD control (11.45 ± 2.08 mmol/L, 6.18 ± 1.92 mmol/L, and 2.89 ± 0.74 mmol/L, respectively). Interestingly, treatment with spores of *E. cristatum* also reduced the TC, TG, and LDL-C levels to 7.86 ± 1.40 mmol/L (*p* < 0.0001), 3.60 ± 1.60 mmol/L (*p* < 0.01), and 1.59 ± 0.36 mmol/L (*p* < 0.0001), respectively. However, neither of the treatments showed a significant effect on serum HDL-C levels. The TC, TG, HDL-C, and LDL-C levels in the livers of golden hamsters for each group were also investigated. As shown in Figure 4, methanol extract of fermented green tea and spore suspension of *E. cristatum* decreased TC (from 0.64 ± 0.17 to 0.45 ± 0.14 and 0.46 ± 0.14 mmol/100 g liver, respectively, both *p* < 0.05) and LDL-C (from 0.57 ± 0.13 to 0.39 ± 0.14 and 0.42 ± 0.10 mmol/100 g liver, with *p* < 0.01 and *p* < 0.05, respectively) levels in liver compared with the HFD control. Both interventions did not show significant effects on TG and HDL-C levels in the liver of golden hamsters.

**Figure 5:**
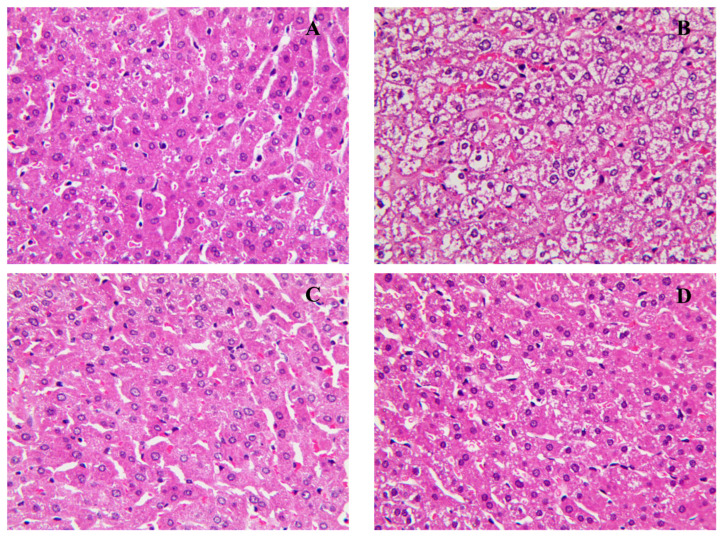
Effects of methanol extract of fermented green tea and spore suspension on fat granules in livers of hamsters for 2 consecutive weeks of intervention. (**A**): normal diet treated group (ND); (**B**): high-fat diet group (HFD): (**C**): high-fat diet with methanol extract of fermented green tea intervention group (MET); (**D**): high-fat diet with the spore intervention group (ST).

To detect the effect of these samples on lipids accumulation in the liver, microscopic observations were performed by the hematoxylin and eosin (H&E) staining method. The liver fat granules in groups treated with methanol extract from fermented green tea and spore suspension of *E. cristatum* were significantly reduced compared with the HFD group (Figure 5). These results indicated that the methanol extract of fermented green tea and spore suspension can both reduce the accumulation of lipid droplets in the hamster’s liver after 2 weeks intervention.

### 3.4. Methanol Extract of Fermented Green Tea and Spore Suspension Improve Diabetes-Related Biomarkers in Serum

**Figure 6:**
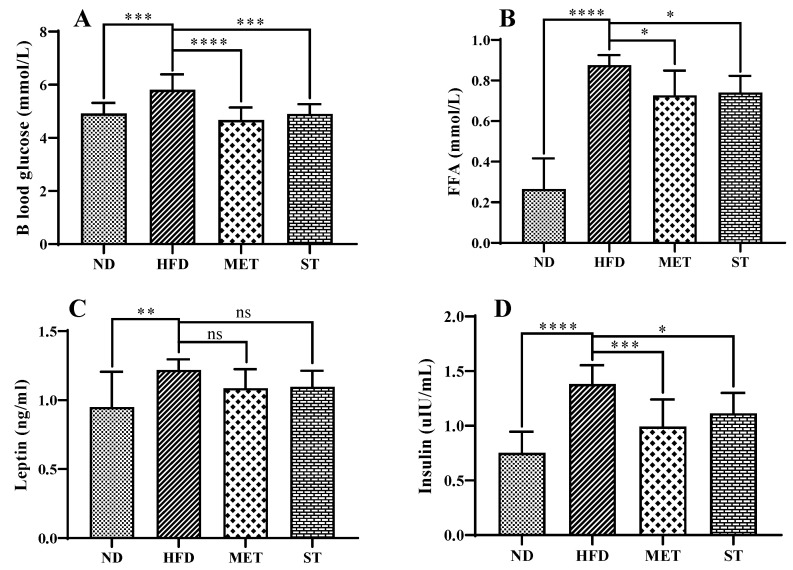
Effects of methanol extract of fermented green tea and spore suspension on diabetes related biomarkers in serum. (**A**): blood glucose; (**B**): FFA; (**C**): Leptin; (**D**): Insulin. Statistical significance of differences between groups was determined using Ordinary one-way ANOVA. Data are expressed as mean ± SD (*n* = 10). * *p* ≤ 0.05; ** *p* ≤ 0.01; *** *p* ≤ 0.001 and **** *p* ≤ 0.0001. “ns” indicates not significant (*p* > 0.05).

The effects of these samples on biomarker levels in serum were evaluated. When hamsters were treated with HFD, the levels of serum glucose, insulin, leptin, and FFA were all significantly increased compared with the ND group, indicating that intake of HFD caused damage to the normal liver function of hamsters (Figure 6). After two weeks of intervention with methanol extract from fermented green tea and spore suspension of *E. cristatum*, the levels of blood glucose, insulin, and FFA were reduced significantly, while the average levels of leptin did not show a significant decrease.

### 3.5. Isolation and Structure Elucidation of Compounds from Spore

**Figure 7:**
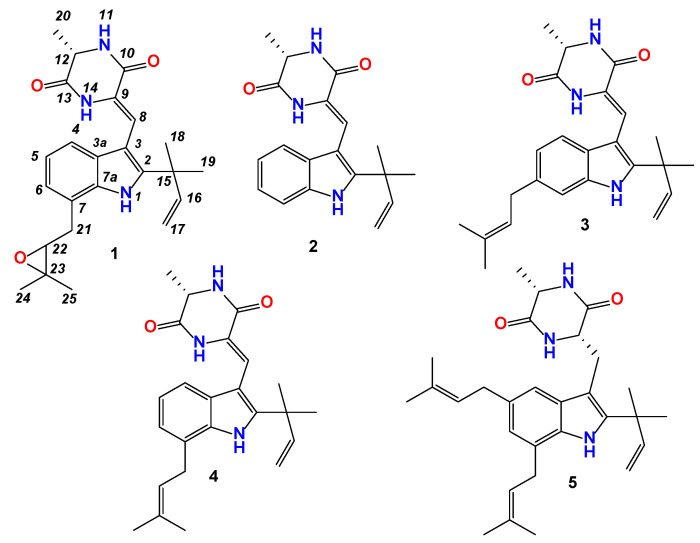
Structures of compounds **1**–**5** isolated from spore.

Both the methanol extract of fermented green tea and spore suspension from fermented wheat reduced the body weight, and serum lipid levels in HFD induced hyperlipidemia model in hamsters. To identify the key components displaying these bioactivities, the chemical components of spore extract were investigated. The spore was extracted by methanol and then purified by preparative HPLC to yield compounds **1**–**5** (Figure 7).

**Figure 8:**
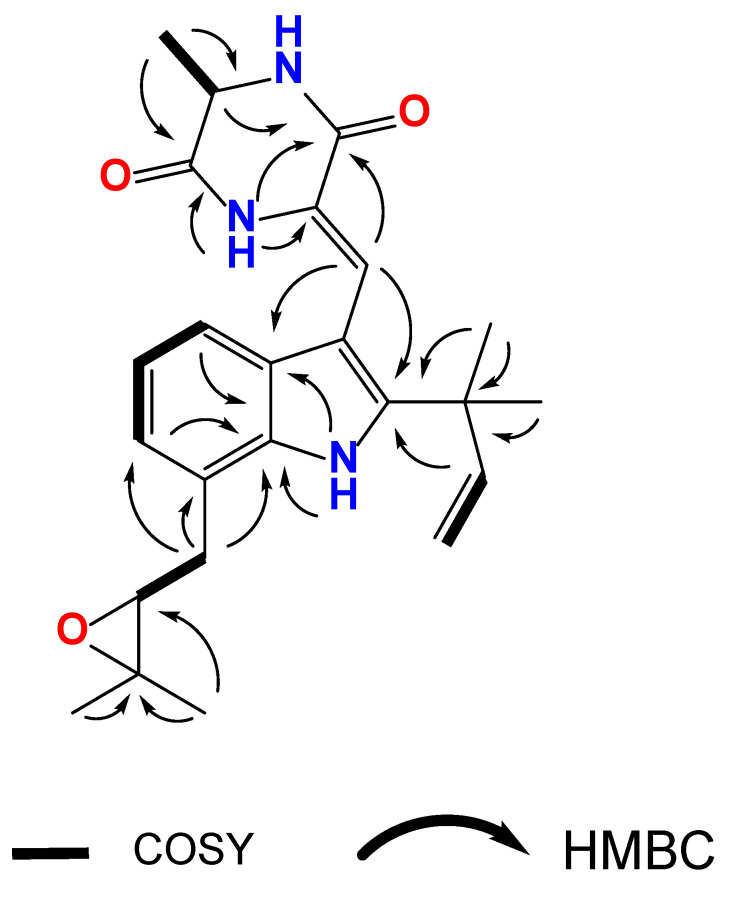
Key ^1^H-^1^H COSY and HMBC correlations for compound **1**.

Compound **1** was isolated as a light yellow amorphous powder. The molecular formula of **1** was determined to be C_24_H_29_N_3_O_3_ based on its high-resolution electrospray ionization mass spectrum (HR-ESIMS) (*m*/*z* [M + H]^+^ 408.2289, calculated for C_24_H_30_N_3_O_3_, 408.2289), accounting for 12 degrees of unsaturation (Figure S1). The ^1^H, ^13^C and HSQC NMR spectra of **1** (Figures S3–S5, Table 1) showed the presence of one doublet methyl group [*δ*_H_ 1.60/*δ*_C_ 21.0 (20-Me)], four singlet methyl groups [*δ*_H_ 1.55/*δ*_C_ 27.4 (18-Me), *δ*_H_ 1.55/*δ*_C_ 27.5 (19-Me), *δ*_H_ 1.51/*δ*_C_ 19.2 (C-24), *δ*_H_ 25.0/*δ*_C_ 140 (C-25)], one sp^3^ methylene group [*δ*_H_ 3.24 and 2.92/*δ*_C_ 33.7 (C-21)], one sp^3^ oxygenated methine [*δ*_H_ 3.01/*δ*_C_ 64.4 (C-22)], one 1,2,3-trisubstituted benzene moiety [*δ*_C_ 126.3 (C-3a), *δ*_H_ 7.19 (d, 8.0)/*δ*_C_ 117.8 (C-4), *δ*_H_ 7.08 (dd, 8.0, 8.0)/*δ*_C_ 121.1 (C-5), *δ*_H_ 6.99 (8.0)/*δ*_C_ 122.7 (C-6), *δ*_C_ 122.2 (C-7), *δ*_C_ 134.2 (C-7a)], one terminal double bond [*δ*_H_ 6.07/*δ*_C_ 144.3 (C-16), *δ*_H_ 5.15 and 5.17/*δ*_C_ 112.9 (C-17)], one olefinic double bond [*δ*_H_ 7.22/*δ*_C_ 112.7 (C-8), *δ*_C_ 112.4 (C-9)], one sp^3^ oxygenated quaternary carbon [*δ*_C_ 60.4 (C-23)], two sp^2^ quaternary carbons [*δ*_C_ 144.6 (C-2), *δ*_C_ 103.2 (C-3)], as well as two carbonyl carbons [*δ*_C_ 160.1 (C-10), *δ*_C_ 165.8 (C-13)]. All these data indicated that compound **1** owns a skeleton of indole-containing diketopiperazine alkaloids. ^1^H-^1^H COSY spectrum (Figure 8 and Figure S6) indicated the moieties of C-4/C-5/C-6, C-12/C-20, and C-21/C-22. In the HMBC spectra, the correlations from H-4 to C-3, C-6, and C-7a, from H-6 to C-7a, from H-NH-1 to C-2, C-3, and C-3a confirmed the indole moiety. The long range HMBC crossing peaks from H_3_-18 and H_3_-19 to C-2, C-15, and C-16 revealed the isopentene moiety (C15/C16/C17/C18/C19) and the connection between C-2 and C-15. The methyl diketopiperazine and the linkage between C-3 and C-8 were suggested by the HMBC correlations from H_3_-20 to C-12 and C-13, from H-NH-14 to C-8, C-9, C-10, C-12, and C-13, and from H-8 to C-2, C-3a, and C-10. The oxygenated side chain was indicated by the HMBC correlations from H_3_-24 and H_3_-25 to C-22 and C-23. C-21 attachment to C-7 was revealed by the HMBC correlations from H-6 to C-21 and from H-21 to C-6, C-7, and C-7a. Combined with molecular formula and chemical shifts for C-22 (*δ*_C_ 64.4) and C-23 (*δ*_C_ 60.4), there should be an epoxy bond between C-2 and C-3. Thus, the structure of **1** was determined as shown in Figure 8 and named variecolorin P. The alanine moiety in **1** was determined as L by comparing its optical rotation (${\left[a\right]}_{D}^{25}$ -30) with the reported analog variecolorin M (${\left[a\right]}_{D}^{25}$ -25) [31].

Four known indole-containing diketopiperazine analogs, (-)-neoechinulin A (**2**) [32], neoechinulin D (**3**) [33], variecolorin G (**4**) [34] and echinulin (**5**) [35] were also isolated and characterized by comparing their molecular weight and NMR data with those reported in the literature. The HPLC peaks of these four compounds as well as compound **1** are labeled in Figure 1.

### 3.6. Compound 1 Attenuated OA-Induced Lipid Accumulation in HepG2 Cells

All of the compounds were assessed using an in vitro cell-based model in which NAFLD was simulated by inducing excessive oleic acid influx into the HepG2 cell line. In the primary screening, with Bodipy staining, compounds **1**, **2**, and **5** attenuated the accumulation of lipids in HepG2 cells with high cell viability at 100, 100, and 25 μg/mL (Table 2). As a new compound, compound **1** was further tested in the Oil Red O assay and showed a significant inhibitory effect against the lipid accumulation with IC_50_ of 0.127 μM.

## 4. Discussion

People with metabolic syndrome normally have conditions including increased blood pressure, abnormal serum levels of blood sugar, cholesterol or triglyceride, and excess body fat. These conditions increase the risk of heart disease, stroke, and type 2 diabetes [36,37]. FBT, a kind of Chinese traditional beverage, is considered to originate in the 16th century (the Ming Dynasty of China) [38]. Chemical investigation of FBT revealed that there are many specific compounds in FBT produced by *E. cristatum* compared with other teas [7,8,9,39,40,41]. The in vivo health benefits of water crude extract of FBT have been investigated [14,15,21,42]. Limited studies focused on the in vivo evaluation of organic solvent extracts of FBT and spore of *E. cristatum* [10,43]. Therefore, we studied the in vivo health benefits of methanol extracts from fermented green tea and spore of *E. cristatum* on a high-fat-diet-induced hyperlipidemia model in hamsters. Furthermore, the chemical components of the spores were analyzed, isolated, and their bioactivity against hyperlipidemia was evaluated.

Our results showed that both the methanol extract from fermented green tea and the spore suspension of *E. cristatum* successfully alleviated the HFD-induced body weight in hamsters. In particular, the ratios of liver weight to body weight were also reduced after two weeks of treatment with interventions compared to HFD control. A study of water extract of FBT on a high-fat diet (HFD)-induced obese mouse assay reported that FBT dramatically ameliorated obesity [14]. *E. cristatum* is non-pathogenic and could survive in the mouse intestine. It can prevent HFD-induced obesity in C57BL/6J mice [12], which is similar to our results. Hyperlipidemia, also known as high cholesterol, means an increase of triacylglycerol (TG), total cholesterol (TC), low-density lipoprotein cholesterol (LDL-C), and a decrease of high-density lipoprotein cholesterol (HDL-C) in blood. HDL absorbs cholesterol from blood and transports it to the liver and improves the metabolism of cholesterol. High levels of HDL lower the risk for cardiovascular diseases [44]. LDL, made up of an outer rim of lipoprotein with a cholesterol center, can accumulate in the walls of blood vessels. High levels of LDL raise the incidence of metabolic diseases [45]. Our study revealed that the methanol extract and spore of *E. cristatum* both can significantly decrease the levels of TC, TG, and LDL-C in serum, and TC and LDL-C in the liver with no significant effects on HDL-C level in serum and liver. A previous study on a hexane extract of FBT [43] reported consistent results with our study. As for the spore of *E. cristatum*, our study reported that a methanol extract of spores of *E. cristatum* showed effects on TG, TC, HDL-C, and LDL-C in serum and liver in hamsters. Non-alcoholic fatty liver disease (NAFLD) is a leading cause of cirrhosis and hepatocellular carcinoma induced by unhealthy diets [46], which is mainly characterized by fat deposition in the liver [47,48]. Liu and co-workers evaluated the hypoglycemic effect of the water extract of spore of *E. cristatum* using a Hep-G2 cell hypoglycemic model and observed the evident increase in glucose consumption [10]. Our results indicated that both the methanol extracts of fermented green tea and spores of *E. cristatum* reduced the accumulation of fatty granules in the livers of hamsters. FBT was reported to have no significant effect on blood glucose homeostasis in HFD mice, while high doses of FBT significantly reduced blood glucose levels in fasting animals [14]. The current study showed that the methanol extract of fermented green tea and spores reduced the blood glucose in hamsters. High insulin levels led to decreased lipid utilization, which will increase the lipid accumulation and aggravate obesity and hyperlipidemia [49]. Zhou and co-workers reported the levels of insulin were decreased after the compound Fuzhuan brick intervention. Compared with glucose, free fatty acids (FFA, or non-esterified fatty acids, NEFA) account for a greater energy flux through the circulation [50,51]. However, a high concentration of FFA induces hepatic toxicity and ectopic lipid deposition, which plays a key role in the pathogenesis of NAFLD [52,53,54]. This study revealed that FBT and spore inhibited the increase of FFA concentration induced by a high-fat diet for the first time. Leptin, a hormone secreted by adipose tissue, can regulate food intake, increase energy release, inhibit the synthesis of adipocytes, and then reduce body weight by participating in the regulation of glucose, fat, and energy metabolism [55]. A high-fat diet feeding increased leptin and affected the body’s glucose and lipid metabolism [56]. A previous study reported that a water extract of dark tea significantly reduced leptin mRNA in the liver, but no significant difference in fasting blood leptin was observed. Similarly, our results showed that two weeks of administration of FBT and spore did not reduce serum leptin. These results demonstrated that real health beneficial components in FBT are mostly contributed by the predominant fungus of *E. cristatum*, while raw tea provided nutrition during the fermentation process. The chemical compositions of FBT were greatly changed during the fermentation process by *E. cristatum* [39,40]. Different classes of metabolites, including polyphenols, phenolic acids, flavones, and their glycosides, terpenoids, alkaloids, steroids, tannins, and fatty acids, have been characterized in FBT [38]. However, limited studies focused on the identification of small molecules with bioactivities related to metabolic syndrome [10,43]. Our study investigated the methanol extract and compounds isolated from the spore of *E. cristatum* with hypolipidemic activity, which indicated the potential developmental value of the spore for functional food and pharmaceutical prospects of compound variecolorin P.

## 5. Conclusions

Our results indicated that the methanol extract of fermented green tea and spores of *E. cristatum* share similar secondary metabolites. Furthermore, both of them improved the hypolipidemic characteristics and obesity in a golden hamster model. Chemical investigation of spores of *E. cristatum* led to the identification of a new indole-containing diketopiperazine alkaloid, variecolorin P (**1**), together with four known analogs, including, (-)-neoechinulin A (**2**), neoechinulin D (**3**), variecolorin G (**4**), and echinulin (**5**). Variecolorin P inhibited the accumulation of lipids in HepG2 cells with IC_50_ of 0.127 μM. Further studies on variecolorin P will reveal the potential value of FBT as a functional food for metabolic diseases.

## Figures and Tables

**Table 1:** ^1^H (500 MHz) and ^13^C NMR (125 MHz) data of Compound **1** (in CDCl_3_).

Position	1		
	δ_C_	δ_H_ (*J* in Hz)	HMBC
1		9.64 s	2, 3, 3a, 7a,
2	144.6		
3	103.2		
3a	126.3		
4	117.8	7.19 d (8.0)	3, 6, 7a
5	121.1	7.08 dd (8.0, 8.0)	3a, 7
6	122.7	6.99 d (8.0)	4, 7a, 21
7	122.2		
7a	134.2		
8	112.2	7.22, s	2, 3a, 10
9	124.0		
10	160.1		
12	51.8	4.29, qd (7.0, 1.5)	10, 13, 21
13	165.8		
14		7.47, s	8, 9, 10, 12, 13
15	39.3		
16	144.3	6.07 dd (17.5, 10.5)	2, 15, 18,19
17a	112.9	5.15 d (17.5)	
17b		5.17 d (10.5)	
18	27.4	1.55 s	2, 15, 16
19	27.5	1.55 s	2, 15, 16
20	21.0	1.60 d (7.0)	12, 13
21a	33.7	2.92 d (15.0, 9.5)	6, 7, 7a, 22, 23
21b		3.24 dd (15.0)	6, 7, 7a, 22, 23
22	64.4	3.01 d (9.5)	21
23	60.4		
24	19.2	1.51 s	
25	25.0	1.40 s	

**Table 2:** Inhibition of lipid accumulation in HepG2 and cytotoxicity of compounds **1**–**5**.

Compound	Concentration (μg/mL)	Inhibitory Rate (%)	Cell Viability (%)
**1**	100	37.30	110.29
**2**	100	6.19	93.97
**3**	100	−67.35	11.15
**4**	100	−15.27	78.06
**5**	25	5.46	94.54

## Data Availability

The data are available from the corresponding author.

## Supplementary Materials

The following supporting information can be downloaded at: https://www.mdpi.com/article/10.3390/nu15061329/s1, Figures S1–S6: HRESIMS, 1D and 2D NMR of compound **1**.
