# Supplementary material for: The Hypolipidemic Characteristics of a Methanol Extract of Fermented Green Tea and Spore of Eurotium cristatum SXHBTBU1934 in Golden Hamsters
Source: Nutrients. 2023 Mar 8;15(6):1329. doi: 10.3390/nu15061329 (PMC10055714; doi:10.3390/nu15061329)
Supplement: Supplementary file 1 [file nutrients-15-01329-s001.zip › nutrients-2215621-supplementary.pdf]

SUPPLEMENTARY MATERIAL

The Hypolipidemic Characteristics of a Methanol Extract of Fermented Green Tea and Spore of *Eurotium cristatum* SXHBTBU1934 in Golden Hamsters

Fuhang Song [1,*], Kai Zhang [1], Jinpeng Yang [2], Annette S. Wilson [3], Caixia Chen [3] and Xiuli Xu [2,*]

Table of Contents

Figure S1. HRESIMS spectrum for **1** 3
Figure S2. ^{1}H NMR spectrum (500 MHz, $CDCl_3$) of **1** 3
Figure S3. ^{13}C NMR spectrum (125 MHz, $CDCl_3$) of **1** 4
Figure S4. HSQC spectrum (500 MHz, $CDCl_3$) of **1** 4
Figure S5. ^{1}H -^{1}H COSY spectrum (500 MHz, $CDCl_3$) of **1** 5
Figure S6. HMBC spectrum (500 MHz, $CDCl_3$) of **1** 5

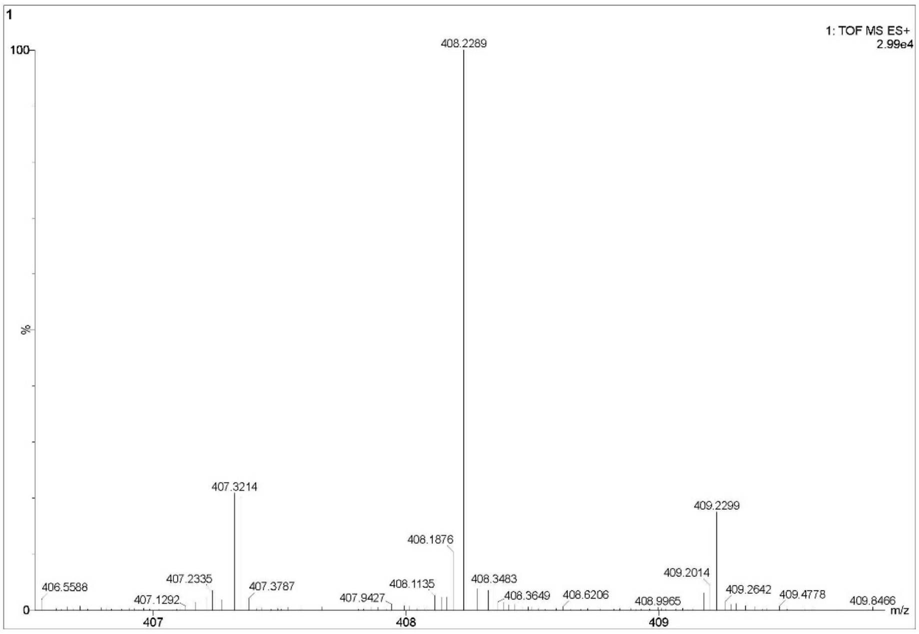

Figure S1. HRESIMS spectrum for **1**.

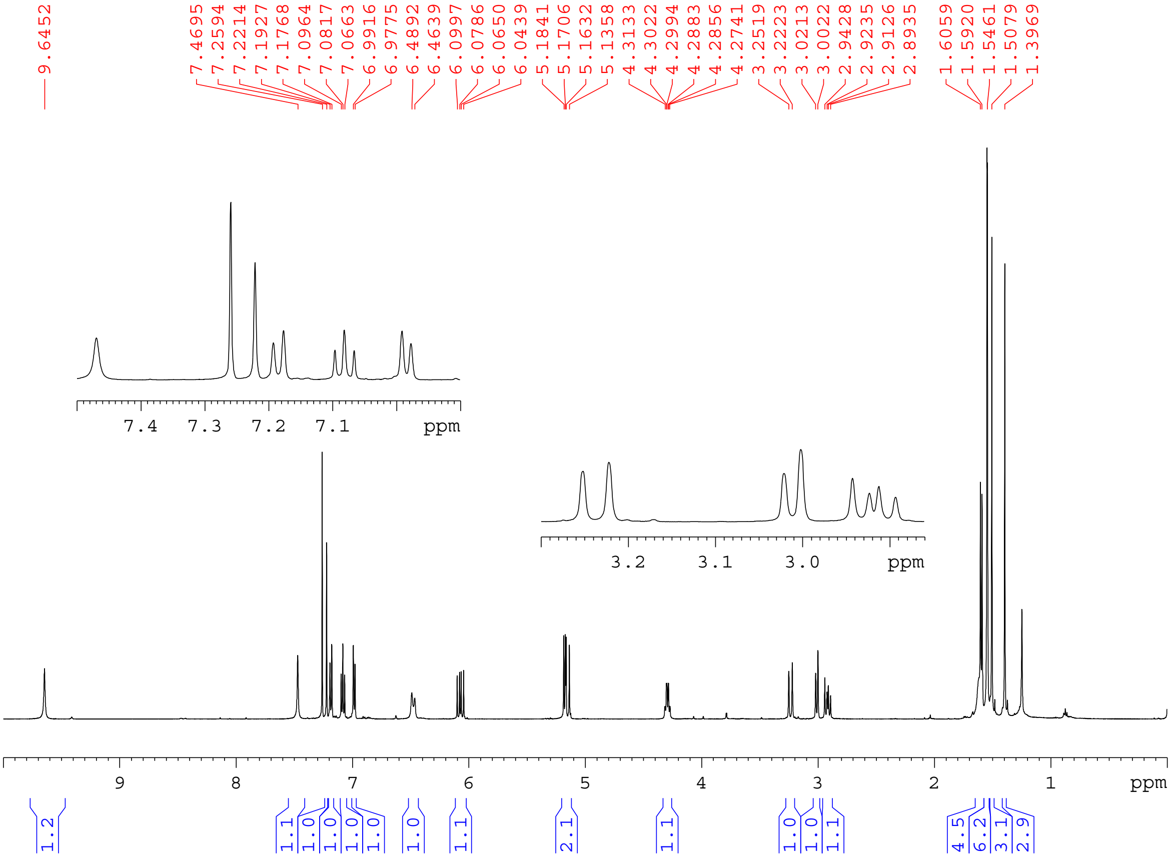

Figure S2. ^{1}H NMR spectrum (500 MHz, $CDCl_3$) of **1**.

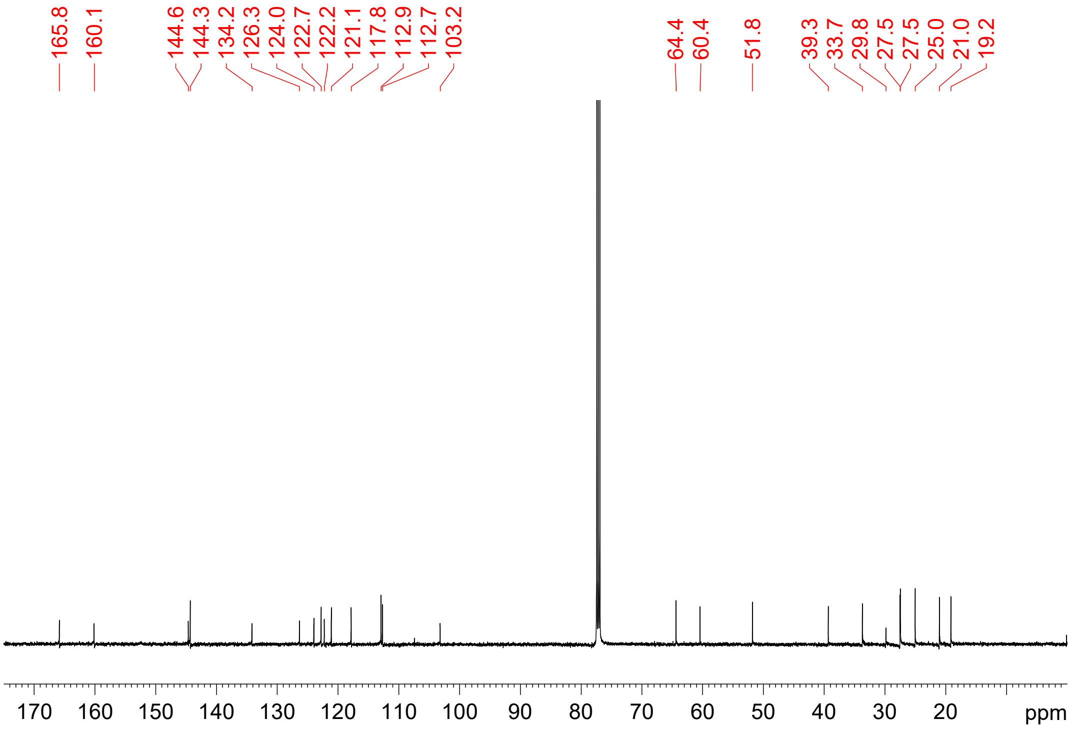

Figure S3. ^{13}C NMR spectrum (125 MHz, $CDCl_3$) of **1**.

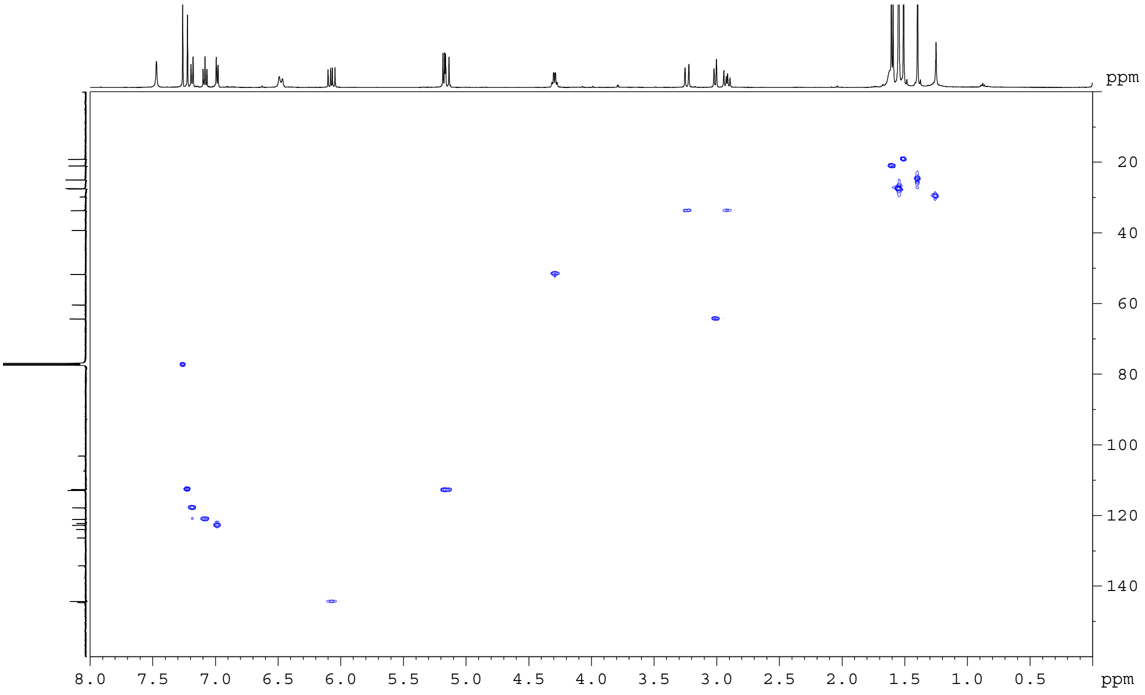

Figure S4. HSQC spectrum (500 MHz, $CDCl_3$) of **1**.

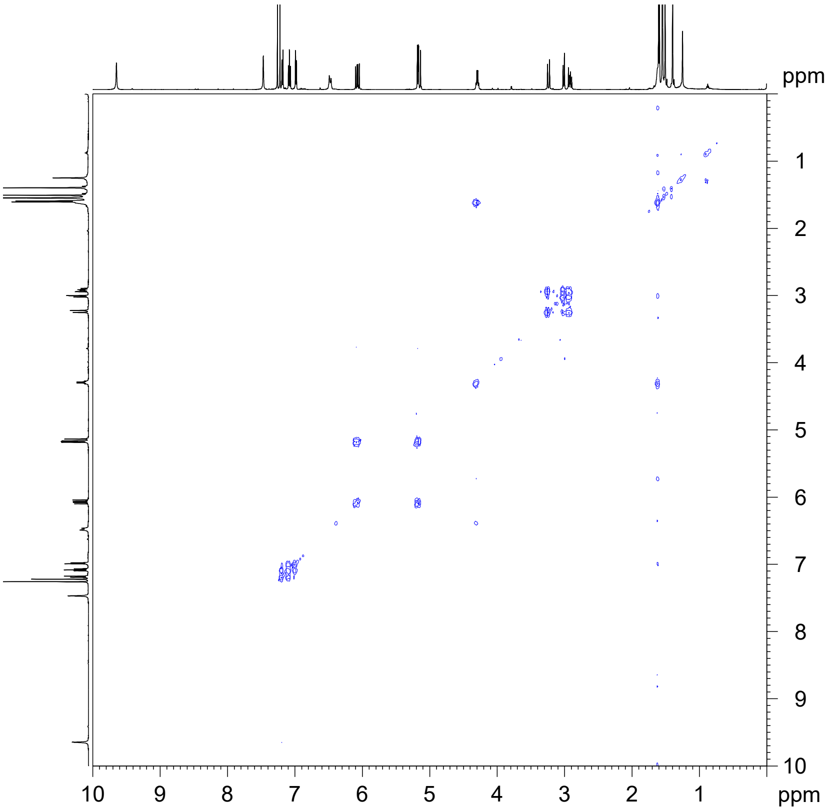

Figure S5. ^{1}H -^{1}H COSY spectrum (500MHz, $CDCl_3$) of **1**.

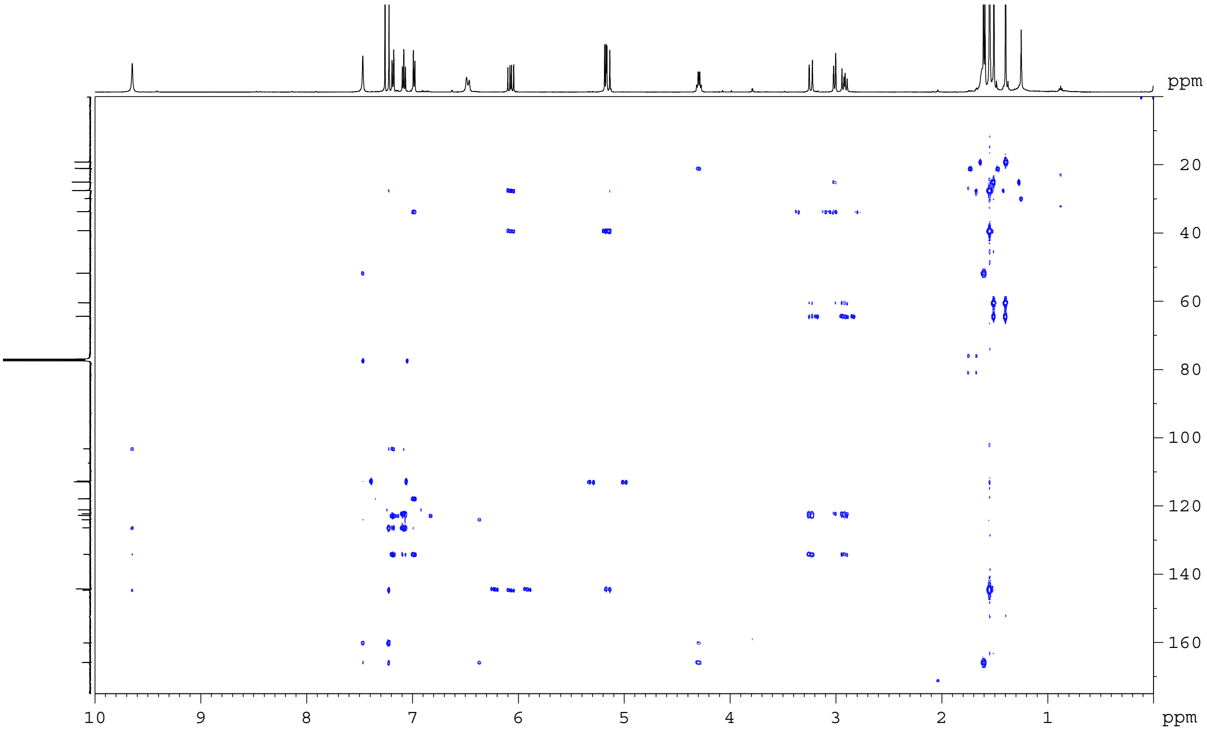

Figure S6. HMBC spectrum (500 MHz, $CDCl_3$) of **1**.
